# Automated Retinal Layer Segmentation Using Graph-based Algorithm Incorporating Deep-learning-derived Information

**DOI:** 10.1038/s41598-020-66355-5

**Published:** 2020-06-12

**Authors:** Zubin Mishra, Anushika Ganegoda, Jane Selicha, Ziyuan Wang, SriniVas R. Sadda, Zhihong Hu

**Affiliations:** 1 Doheny Image Analysis Laboratory, Doheny Eye Institute, Los Angeles, CA 90033 USA; 2 The University of California, Los Angeles, CA 90095 USA

**Keywords:** Prognostic markers, Information technology

## Abstract

Regular drusen, an accumulation of material below the retinal pigment epithelium (RPE), have long been established as a hallmark early feature of nonneovascular age-related macular degeneration (AMD). Advances in imaging have expanded the phenotype of AMD to include another extracellular deposit, reticular pseudodrusen (RPD) (also termed subretinal drusenoid deposits, SDD), which are located above the RPE. We developed an approach to automatically segment retinal layers associated with regular drusen and RPD in spectral domain (SD) optical coherence tomography (OCT) images. More specifically, a shortest-path algorithm enhanced with probability maps generated through a fully convolutional neural network was used to segment drusen and RPD, as well as 11 retinal layers in SD-OCT volumes. This algorithm achieves a mean difference that is within the subpixel accuracy range drusen and RPD, alongside the other 11 retinal layers, highlighting the high robustness of this algorithm for this dataset. To the best of our knowledge, this is the first report of a validated algorithm for the automated segmentation of the retinal layers including early AMD features of RPD and regular drusen separately on SD-OCT images.

## Introduction

Spectral-domain optical coherence tomography (SD-OCT) is a three-dimensional (3-D), *in vivo* imaging technique that permits direct visualization of retinal morphology and architecture^1^.

In the setting of retinal or optic nerve disease, the retinal layer thickness may be affected either locally or globally, depending on the specific disease. In terms of vision and blindness in the United States, the three most important diseases are age-related macular degeneration (AMD), diabetic retinopathy, and glaucoma^2–4^. AMD is the leading cause of blindness in people aged 65 and older in the western world^5^. Although effective treatments are now available for patients with neovascular AMD (choroidal neovascularization, CNV), many of these patients appear to eventually lose vision due to the development of geographic atrophy (GA). Several agents have been evaluated, including in Phase 3 clinical trials, hoping to slow the progression of GA, but thus far there is no proven, effective treatment. Some researchers have suggested that earlier intervention before the development of irreversible atrophy might be a preferable strategy.

Reticular pseudodrusen and regular drusen are now both accepted as features that are commonly present in early and intermediate AMD, before the development of late features such as CNV or GA. Drusen commonly appear as tiny yellow or white deposits at the level of the Bruch’s membrane on color fundus images, but they are visualized more easily by SD-OCT. The importance of drusen in the diagnosis and progression of AMD has been established in a number of studies^6–8^. Advances in imaging over the past two decades, including in infrared reflectance (IR) and autofluoresence, have revealed another distinct phenotype, known as reticular pseudodrusen (RPD), or subretinal drusenoid deposits (SDD). RPD are strongly associated with the development of late AMD, including Type 3 (intraretinal) NV (also known as retinal angiomatous proliferation or RAP), outer retinal atrophy (ORA) and GA. RPD, which can appear as yellowish interlacing networks in color fundus images, are also better visualized using infrared imaging, blue light fundus autofluorescence (FAF), or SD-OCT. Studies correlating SD-OCT and confocal scanning laser ophthalmoscopy have shown that RPD appear to correspond to subretinal deposits, located internal to the RPE, in contrast to traditional drusen, which are located external to the RPE^9^. As multiple longitudinal studies have revealed that RPD are strong predictors for progression to GA^9–13^, interest in understanding the role that RPD play in the pathogenesis of AMD has grown. Quantifying and monitoring the progression of these features of AMD are of interest for understanding the evolution of AMD. Manual segmentation of these AMD features and associated alterations in retinal layers in volumetric SD-OCT images, however, is tedious, time-consuming, and impractical for large studies. While a system for automatically detecting the AMD biomarkers and multiple retinal layers is attractive, automated detection is not trivial due to the complexity of the layer structures relatively low image contrast of OCT, and the disturbance of the retinal layers due to the AMD features/biomarkers, for instance the regular drusen and RPD. RPD are particularly difficult to identify and segment as the borders of these lesions may be poorly demarcated. As such, to the best of our knowledge, there have been no reported validated algorithms for automated segmentation of RPD. We have previously reported semiautomated segmentation of outer retinal layers in eyes with RPD^14^, but segmentation failures precluded accurate quantification of RPD themselves.

Approaches to the problem of automated segmentation in SD-OCT volumes have included application of shortest path frameworks^15,16^, active contour modeling^17^, graph search frameworks^18–21^, and deep learning methods^22–26^. Of these approaches, graph-based approaches have generally performed best. However, graph-search frameworks are slow and shortest-path frameworks do not have interaction constraints. The algorithm discussed in this paper is based on the shortest path framework and makes use of a fully convolutional neural network (i.e. the U-Net^22–24^) to enhance performance. Our major contribution with this algorithm is the automatic segmentation of RPD in SD-OCT images. We are additionally able to automatically segment regular drusen and explore the applicability of this method in the automatic segmentation of 11 retinal surfaces, including the various retinal bands, in SD-OCT images. To the best of our knowledge, this is the first report of a validated algorithm for the automated segmentation of the retinal layers including early AMD features of RPD and regular drusen separately on SD-OCT images.

## Methods

### Overview

**Figure 1:**
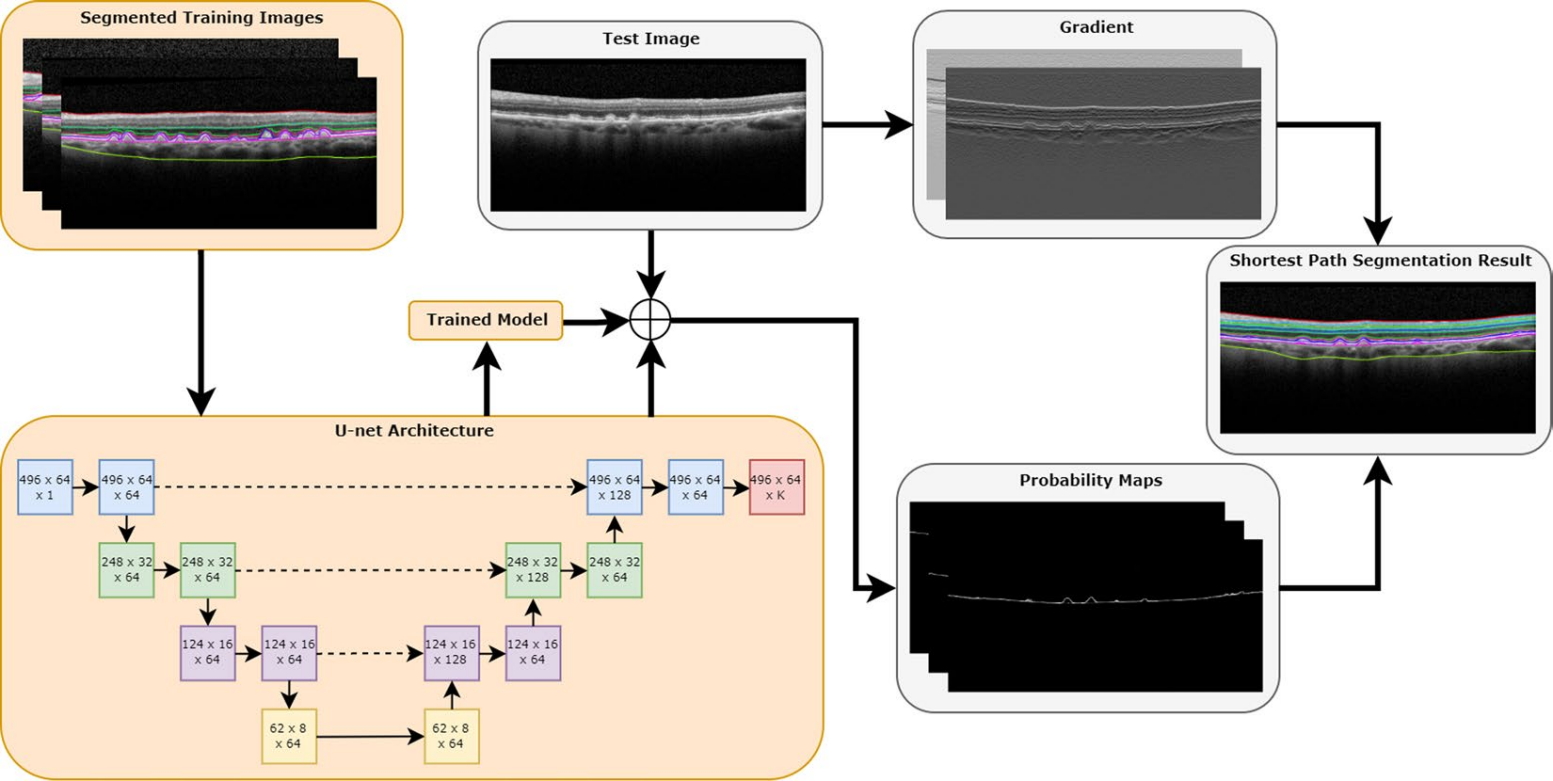
Overview of the DL-SP approach for segmentation of retinal layers, where orange boxes represent training steps and gray boxes represent evaluation steps. There was no overlap between training and testing data.

Overall, the Deep Learning – Shortest Path (DL-SP) algorithm as described in this report is a multistage shortest path segmentation approach which is applied to delineate RPD and drusen and can further be applied to segment 11 retinal surfaces in SD-OCT. To be more computationally efficient, instead of performing the retinal layers’ segmentation in OCT volumes using the 3D graph-based approach as in our previous work, the DL-SP algorithm is performed on 2D OCT B-scan images. The pixel to pixel edge weights used in the shortest path algorithm were calculated using the normalized gradient in the z-direction (inverted to measure dark-to-bright or bright-to-dark intensity transition depending on the layer) in combination with probability maps generated from a deep learning fully convolutional neural network based on the U-Net architecture. Figure 1 shows an overview of the method.

### Imaging dataset

45 eyes from 42 subjects were identified from patients at the Doheny-UCLA Eye Centers. Of these eyes, 25 were diagnosed with AMD, and the remaining 20 eyes were healthy. 16 eyes diagnosed with AMD were used to train neural networks to identify RPD and drusen. The healthy eyes were used to train the segmentation of layers largely unaffected by the presence of RPD and drusen. All images were de-identified according to Health and Insurance Portability and Accountability Act Safe Harbor prior to analysis. Ethics review and institutional review board approval from the University of California – Los Angeles were obtained. The research was performed in accordance with relevant guidelines/regulations, and informed consent was obtained from all participants.

Each subject underwent volume OCT imaging using a Heidelberg Spectralis (Heidelberg Engineering, Heidelberg, Germany) SD-OCT. All volume scans consisted of macular cube scan patterns. The image scan dimensions for eyes diagnosed with AMD are 496 (depth) × 1024 (A-scans) × 49 (B-scans) pixels, 496 (depth) × 1536 (A-scans) × 61 (B-scans) pixels, 496 (depth) × 512 (A-scans) × 49 (B-scans) pixels, 496 (depth) × 512 (A-scans) × 97 (B-scans) pixels, or 496 (depth) × 768 (A-scans) × 49 (B-scans) pixels. All images were cropped, stretched, or both in order to resize images to a standard width of 1024 and height of 496. After cropping, the average physical dimensions for these scans are 1.93 mm (depth) × 6.27 mm (A-scans) × 5.82 mm (B-scans). For the healthy eyes, the image scan dimensions are 496 (depth) × 1024 (A-scans) × 37 (B-scans) pixels. The average physical dimensions for these scans are 1.92 mm (depth) × 5.90 mm (A-scans) × 4.57 mm (B-scans). The pixel depth was 8 bits in grayscale. To provide consistency for the quantitative analysis of the retinal layer properties among different volume scans, all the right eye scans were horizontally flipped in the x-direction.

### Shortest path

Shortest path segmentation is one of the fully automatic approaches to segmenting retinal layers that DL-SP builds on^27^. The shortest path algorithm for retinal segmentation is a graph-based approach. For each SD-OCT B-scan image, each pixel acts as a node, with edges reaching out to each of its eight neighboring pixels. With this representation, pathways crossing the image can be thought of as sets of connected edges. By assigning each edge a weight, path preferences can be formed. Once weights are assigned, Dijkstra’s algorithm is used to identify the path between two pixels with the lowest total weight. To ensure that each segmented layer crosses the entire image, two additional columns of pixels are added as buffer nodes with minimal weights assigned to their edges, and the top-left and bottom-right pixels are chosen to be the start and end nodes, respectively, for Dijkstra’s algorithm.

Edge weights are determined by changes in pixel intensity. Each retinal layer to be segmented is associated with a change from high to low or low to high pixel intensity. Therefore, two adjacency matrices are created, one using a normalized intensity gradient and the other using its inverse. The edge weights are calculated using the following equation:

1 $$\begin{array}{c}{W}_{a,b}=2-({G}_{a}+{G}_{b})+{W}_{min}\end{array}$$

where *W*_*a,b*_ is the weight assigned to the edge connecting pixels *a* and *b*, *G*_*a*_ and *G*_*b*_ are the vertical gradients of the image at pixels *a* and *b*, and *W*_*min*_ is the minimal weight used in the two buffer columns.

Layers are segmented in order of prominence of changes of intensity; as such, the inner limiting membrane (ILM) and inner-outer segment junction (IS-OS) are the first layers found. After one layer is successfully segmented, it is used to limit the region of interest that is searched for the next layers. This aids in segmenting layers that exhibit less contrast than the ILM and IS-OS. In the DL-SP approach, probability maps generated by a convolutional neural network for each layer boundary are incorporated into this approach, changing the edge weight calculation and the determination of regions of interest for certain layers (see Section “Deep learning - shortest path”).

### Deep learning

The second approach to retinal segmentation DL-SP builds upon uses a deep learning fully convolutional neural network to determine the retinal layers^24^. This neural network is a U-Net, a state-of-the-art deep learning algorithm for semantic segmentation relying on encoder-decoder type network architecture^22^. U-Net does not use fully connected layers and does not require sliding windows, resulting in faster performance that maintains precision even with smaller datasets^22^. The network consists of a contracting path of encoder blocks followed by and expansive (upsampling) path of three decoder blocks. Each encoder block consists of layers of convolution, batch normalization, rectified linear unit (ReLU), and pooling. Each decoder block consists of layers of unpooling, concatenation, convolution, batch normalization, and ReLU. The convolution kernels for all the encoder blocks were maintained at 7 × 3. Finally, a convolutional layer with a 1 × 1 kernel brings the final channel count down to the number of layers being segmented, and a softmax layer calculates the probabilities for the assignment of each pixel into a layer. The U-Net is optimized through the following functions:

2 $$\begin{array}{c}CE=\sum _{x\in \Omega }w(x){g}_{l}(x)\log ({p}_{l}(x))\end{array}$$

3 $$\begin{array}{c}Dice=1-\frac{2{\sum }_{x\in \Omega }{p}_{l}(x){g}_{{\rm{l}}}(x)}{{\sum }_{x\in \Omega }{{\rm{g}}}_{{\rm{l}}}^{2}(x)+{\sum }_{x\in \Omega }{p}_{{\rm{l}}}^{2}(x)}\end{array}$$

4 $$\begin{array}{c}Overall=CE+Dice+\lambda {\Vert {\bf{W}}\Vert }_{F}^{2}\#\end{array}$$

where *CE* is the cross-entropy loss, *Dice* is the Dice loss, *Overall* is the overall loss, *w*(*x*) is the weight assigned to pixel $x\in \Omega$, *g*_*l*_(*x*) is the ground truth probability of a pixel for layer *l*, *p*_*l*_(*x*) is the assigned probability of a pixel for layer *l*, *λ* is the weight decay, and **W**_*F*_ is the Frobenius norm on the weights of the U-net^24^.

For DL-SP, of the eyes diagnosed with AMD, 16 eyes (831 2D B-scans) were randomly selected to be used for training. The remaining 9 eyes (512 2D B-scans) were used for testing. The loss function is optimized using stochastic mini-batch gradient descent in mini batches of size = 8 slices. Slices were formed by cutting images into nonoverlapping sections of width 64. At the start of training, the learning rate is set to 0.1 and is reduced by an order of magnitude every 20 epochs for a total of 60 epochs. Training was performed at a momentum of 0.95 with a weight decay of 0.0001. Pixel weights were assigned as 1 for outside the targeted layers, 6 for the targeted layers, and 15 for the targeted layer boundaries. Our U-Net was implemented in MATLAB R2017a and run on a desktop PC with an Intel i7-7800X CPU, 16 GB NVIDIA Quadro P5000 GPU, and 16 GB RAM.

**Figure 2:**
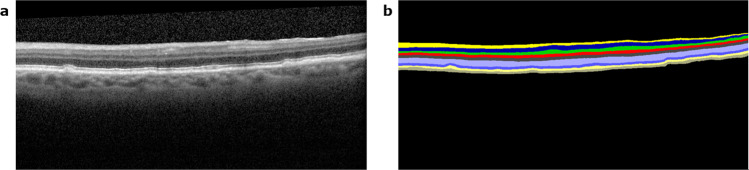
Results of the deep learning fully convolutional neural network, from which probability maps for the boundaries between layers can be calculated. (**a**) shows the original B-scan. (**b**) visualizes the layer assignment generated by the neural network.

**Figure 3:**
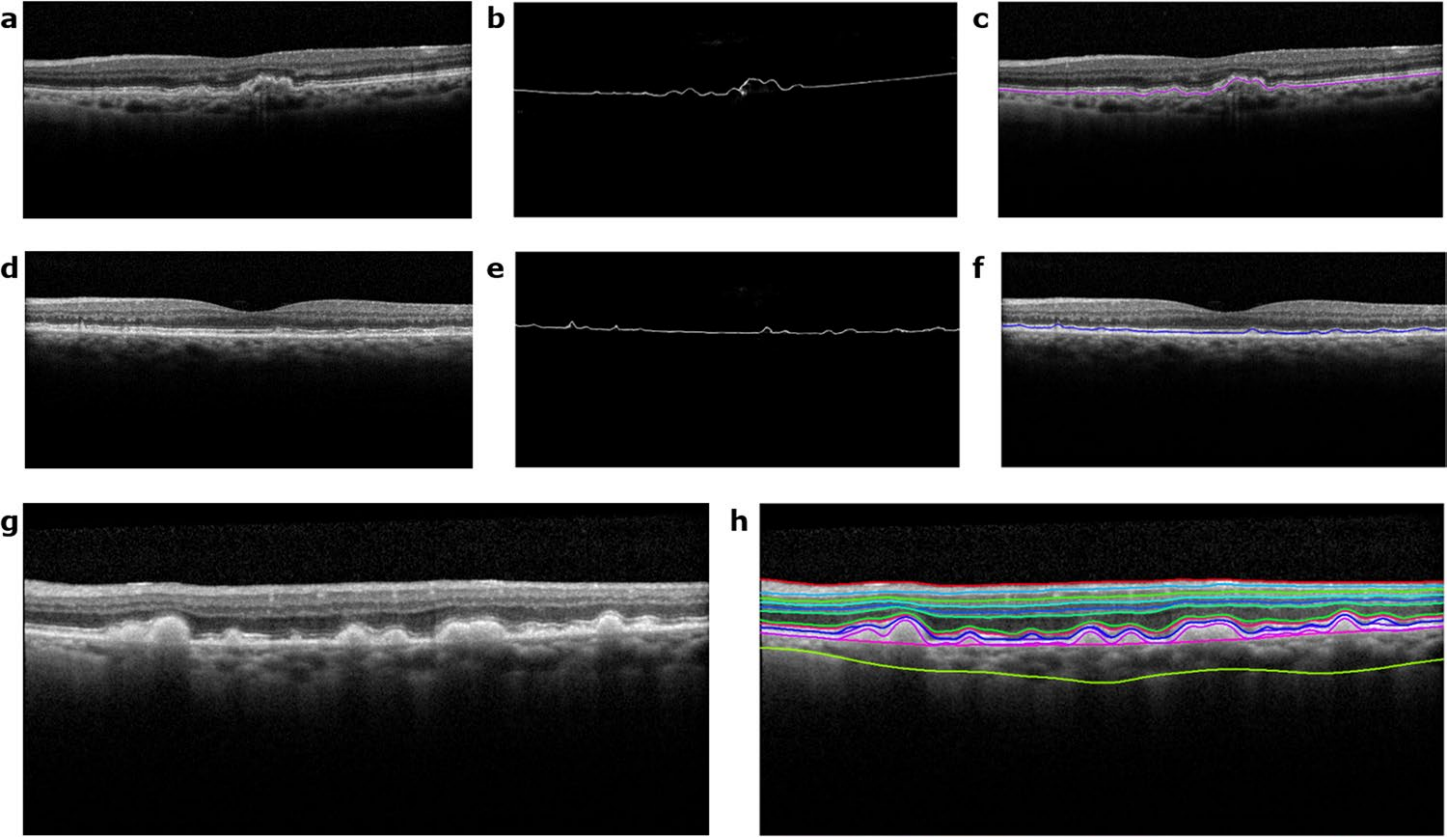
Segmentation of drusen, RPD, and eleven retinal layers on SD-OCT B-scans. (**a**,**d**,**g**) are the original B-scans for the segmentations shown in (**c**,**f**,**h**) respectively. The drusen segmentation shown in (**c**) arises from the deep learning probability map shown in (**b**) and the RPD segmentation shown in (**f**) arises from the deep learning probability map shown in (**e**) The layers in order from top to bottom are the ILM, NF-GC, GC-IP, IP-IN, IN-OP, OP-ON, ELM, IS-OS, RPD, inner RPE, drusen, outer RPE, and C-S.

Each pixel location was assigned a probability for each class in the label space L = {l} = {1, · · ·, K} for K classes, including RPD and drusen (Fig. 2). Using these layer probabilities, probability maps for the layer boundaries were found following

5 $$\begin{array}{c}{P}_{i,i+1}=1-|\mathop{\sum }\limits_{j=1}^{i}{P}_{j}-\mathop{\sum }\limits_{j=i+1}^{K}{P}_{j}|\end{array}$$

where ${P}_{i,i+1}$ is the probability of a pixel being part of the boundary between layer classes *i* and i + 1, and *P*_*j*_ is the probability of a pixel being part of layer class *j*, as assigned by the neural network. A visualization of these probability maps is shown in Fig. 3. For layers demonstrating low image contrast, comparatively, more focused neural networks were trained and applied to enhance the accuracy of the boundaries found by using prior segmentation to create an upper and lower boundary on which a neural network could train and be utilized on. Specifically, this was done for the choroid-sclera (C-S) boundary and the ganglion cell-inner plexiform (GC-IP) junction.

### Deep learning – shortest path

The Deep Learning – Shortest Path algorithm makes use of both the above approaches. The first step in the algorithm is generating the probability maps as described in Section 2.4. These probability maps are incorporated into the calculation of weights of pixel to pixel edges in a variation of equation (1):

6 $$\begin{array}{c}{W}_{a,b}=2({w}_{g}+{w}_{p})-{w}_{g}({G}_{a}+{G}_{b})-{w}_{p}({P}_{a}+{P}_{b})+{W}_{min}\end{array}$$

where *w*_*g*_ is the weight given to the gradient, *w*_*p*_ is the weight given to the probability map, and *P*_*a*_ and *P*_*b*_ are the probability map values at pixels *a* and *b*. With DL-SP, the importance placed on the vertical gradient and the probability can be varied by changing *w*_*g*_ and *w*_*p*_. It is possible for these weightings to be different for each layer segmented. Because weights are calculated uniquely for each layer with this method, an adjacency matrix is required for each layer to be segmented, as opposed to the two required for the aforementioned shortest path method.

The segmentations of the layers were performed in the following sequence: the internal limiting membrane (ILM), inner-outer segment (IS-OS) junction, outer retinal pigment epithelium (RPE), drusen, inner RPE, retinal pseudodrusen (RPD), inner nuclear-outer plexiform (IN-OP) junction, nerve fiber-ganglion cell (NF-GC) junction, inner plexiform-inner nuclear (IP-IN) junction, outer plexiform-outer nuclear (OP-ON) junction, external limiting membrane (ELM), ganglion cell-inner plexiform (GC-IP) junction, and choroid-sclera (C-S) junction.

As in the original shortest-path algorithm, regions of interest for each layer are narrowed down using the layers previously segmented. However, in the cases of drusen and RPD, the region of interest is further limited using the probability maps generated for those layers (*P*_*drusen*_ and *P*_*RPD*_ by the previously established notation). The probability maps were binarized, and the groups of pixels marked as RPD and drusen were isolated. RPD smaller than 5 pixels and drusen smaller than 11 pixels were removed from consideration. Regions of interest for drusen and outer RPE were then established by taking the first and last marked pixels in each column of the drusen probability map, respectively, and the same was done for RPD and inner RPE with the marked pixels of the RPD probability map. Connectivity was then ensured between these probability map-derived regions of interest and the previous, broader region of interest. Through this process, the region of interest for segmentation at the site of the AMD features is much narrowed. This, combined with the calculated boundary layer probability maps, allowed for accurate layer segmentation for layers exhibiting the early AMD pathology.

To summarize, a deep learning fully convolutional neural network was used to calculate probability maps for layer junctions and identify RPD and drusen regions of interests. A shortest path algorithm with weights determined by these probability maps and the gradient of the SD-OCT scan determined the layer segmentation. Finally, a cubic spline fitting was applied to smooth the segmented layers. The fitting was applied mainly to correct the small localized segmentation errors, most notably in regions of vessel shadows cast by overlying large retinal vessels.

### Use of human participants

Ethics review and institutional review board approval from the University of California – Los Angeles were obtained. The research was performed in accordance with relevant guidelines/regulations, and informed consent was obtained from all participants.

## Results

To evaluate the accuracy of the automated layer segmentation, the layers for each case were manually traced by a certified OCT grader from the Doheny Image Reading Center, who was masked to the automated results. No refinement was performed of the automated segmentations before the comparison with the manual tracing. Because we desired a high-level of precision, even a single pixel discrepancy in the position of the border at any location was deemed to constitute a discrepancy. The reading center medical director re-reviewed and confirmed the gradings and boundaries for each case. The accuracy of the automated surface segmentation was evaluated in terms of the mean and absolute mean differences in the z-position between the segmented layers and the corresponding manually traced layers from the expert grader. For the mean differences, a negative value indicated that a segmented surface was located above the corresponding manually traced surface, and a positive value indicated that a segmented surface was located below the corresponding manually traced surface.

Table 1 shows the mean and absolute average border position differences of 7 layers: ILM, OP-ON, ELM, IS-OS, inner RPE, outer RPE, and the C-S junction from the automated segmentation for a 1:1 probability map to gradient weighting. It also shows the mean and absolute average border position differences for RPD and drusen. The remaining 4 layers, NF-GC, GC-IP, IP-IN, IN-OP, have been segmented to subvoxel accuracy by our group previously^21^. We believe the DL-SP algorithm will result in similar accuracy. Tables 2–5 show the mean and absolute average border position differences for various other pixel to pixel edge weighting schemes. In Fig. 3, RPD, drusen, and the segmented 11 layers are shown overlaid on SD-OCT images.

**Table 1:** Mean and absolute mean border position differences of the automated and manual segmentations on a 1:1 probability map to gradient weighting scheme (512 2D B-scans).

Layers	Mean (pixels)	Mean (μm)	Absolute Mean (pixels)	Absolute Mean (μm)
ILM	−0.70 ± 0.95	−2.74 ± 3.68	0.89 ± 0.78	3.45 ± 3.03
OP-ON	−0.59 ± 2.71	−2.29 ± 10.53	1.67 ± 2.21	6.49 ± 8.60
ELM	−0.47 ± 2.66	−1.81 ± 10.37	2.02 ± 1.80	7.84 ± 7.02
IS-OS	−0.60 ± 1.25	−2.34 ± 4.87	1.01 ± 0.95	3.92 ± 3.72
Inner RPE	−0.58 ± 1.88	−2.27 ± 7.31	1.42 ± 1.36	5.53 ± 5.29
Outer RPE	0.02 ± 1.84	0.07 ± 7.16	1.14 ± 1.44	4.44 ± 5.62
C-S	−0.12 ± 13.37	−0.48 ± 52.04	9.31 ± 9.60	36.22 ± 37.37
Drusen	−0.41 ± 1.97	−1.58 ± 7.66	1.30 ± 1.53	5.05 ± 5.97
RPD	−0.75 ± 1.99	−2.92 ± 7.74	1.53 ± 1.47	5.97 ± 5.74

**Table 2:** Mean and absolute mean border position differences of the automated and manual segmentations based only on gradient (512 2D B-scans).

Layers	Mean (pixels)	Mean (μm)	Absolute Mean (pixels)	Absolute Mean (μm)
ILM	0.96 ± 3.74	3.72 ± 14.54	1.17 ± 3.68	4.56 ± 14.30
OP-ON	4.87 ± 8.37	18.96 ± 32.55	5.49 ± 7.98	21.36 ± 31.04
ELM	3.14 ± 3.27	12.24 ± 12.72	3.39 ± 3.01	13.19 ± 11.72
IS-OS	0.31 ± 1.97	1.21 ± 7.66	1.14 ± 1.63	4.44 ± 6.35
Inner RPE	1.01 ± 3.15	3.94 ± 12.27	2.68 ± 1.95	10.43 ± 7.58
Outer RPE	0.93 ± 3.17	3.62 ± 12.32	1.98 ± 2.64	7.71 ± 10.28
C-S	−12.69 ± 21.34	−49.39 ± 83.03	17.59 ± 17.52	68.46 ± 68.16
Drusen	0.79 ± 3.17	3.07 ± 12.32	2.10 ± 2.50	8.17 ± 9.73
RPD	1.02 ± 3.25	3.96 ± 12.64	2.76 ± 1.99	10.75 ± 7.74

**Table 3:** Mean and absolute mean border position differences of the automated and manual segmentations based only on probability map (512 2D B-scans).

Layers	Mean (pixels)	Mean (μm)	Absolute Mean (pixels)	Absolute Mean (μm)
ILM	−0.79 ± 0.95	−3.07 ± 3.68	0.95 ± 0.79	3.68 ± 3.07
OP-ON	−0.65 ± 2.74	−2.52 ± 10.66	1.68 ± 2.26	6.54 ± 8.78
ELM	−0.49 ± 2.93	−1.90 ± 11.42	2.05 ± 2.16	7.97 ± 8.40
IS-OS	−0.67 ± 1.90	−2.61 ± 7.39	1.09 ± 1.69	4.26 ± 6.58
Inner RPE	−0.61 ± 1.90	−2.38 ± 7.40	1.44 ± 1.38	5.61 ± 5.38
Outer RPE	0.00 ± 1.83	−0.01 ± 7.13	1.15 ± 1.43	4.47 ± 5.56
C-S	0.76 ± 12.25	2.94 ± 47.68	9.23 ± 8.09	35.93 ± 31.48
Drusen	−0.43 ± 1.98	−1.69 ± 7.70	1.31 ± 1.54	5.10 ± 6.00
RPD	−0.79 ± 2.01	−3.07 ± 7.82	1.56 ± 1.50	6.05 ± 5.82

**Table 4:** Mean and absolute mean border position differences of the automated and manual segmentations on 1:2 probability map to gradient weighting scheme (512 2D B-scans).

Layers	Mean (pixels)	Mean (μm)	Absolute Mean (pixels)	Absolute Mean (μm)
ILM	−0.62 ± 0.95	−2.39 ± 3.69	0.83 ± 0.76	3.25 ± 2.97
OP-ON	−0.54 ± 2.77	−2.11 ± 10.79	1.67 ± 2.28	6.51 ± 8.86
ELM	−0.48 ± 2.66	−1.88 ± 10.36	2.02 ± 1.80	7.87 ± 7.00
IS-OS	−0.56 ± 1.25	−2.19 ± 4.85	0.98 ± 0.95	3.83 ± 3.70
Inner RPE	−0.56 ± 1.92	−2.20 ± 7.47	1.44 ± 1.39	5.60 ± 5.40
Outer RPE	0.06 ± 1.79	0.21 ± 6.96	1.14 ± 1.38	4.42 ± 5.38
C-Sv	−0.13 ± 13.39	−0.51 ± 52.12	9.32 ± 9.62	36.27 ± 37.43
Drusen	−0.37 ± 1.93	−1.45 ± 7.51	1.29 ± 1.49	5.00 ± 5.79
RPD	−0.73 ± 2.03	−2.84 ± 7.90	1.55 ± 1.50	6.03 ± 5.84

**Table 5:** Mean and absolute mean border position differences of the automated and manual segmentations on 2:1 probability map to gradient weighting scheme (512 2D B-scans).

Layers	Mean (pixels)	Mean (μm)	Absolute Mean (pixels)	Absolute Mean (μm)
ILM	−0.75 ± 0.95	−2.91 ± 3.68	0.92 ± 0.78	3.56 ± 3.05
OP-ON	−0.61 ± 2.66	−2.38 ± 10.36	1.66 ± 2.17	6.47 ± 8.44
ELM	−0.46 ± 2.66	−1.78 ± 10.37	2.01 ± 1.81	7.83 ± 7.02
IS-OS	−0.62 ± 1.25	−2.42 ± 4.88	1.02 ± 0.96	3.98 ± 3.72
Inner RPE	−0.60 ± 1.88	−2.33 ± 7.31	1.43 ± 1.36	5.56 ± 5.29
Outer RPE	0.02 ± 2.00	0.07 ± 7.79	1.15 ± 1.63	4.49 ± 6.36
C-S	−0.09 ± 13.36	−0.34 ± 51.97	9.31 ± 9.58	36.23 ± 37.27
Drusen	−0.42 ± 1.98	−1.63 ± 7.69	1.30 ± 1.54	5.08 ± 6.00
RPD	−0.77 ± 1.99	−3.00 ± 7.73	1.54 ± 1.47	5.99 ± 5.73

## Discussion and Conclusions

In this paper, an automated approach was developed to segment RPD and regular drusen separately on SD-OCT images. The approach can be extended to segment a further 11 retinal surfaces on SD-OCT images. The mean difference is within a subpixel accuracy range for all layers, highlighting the robustness of this algorithm. Particularly, the algorithm achieved mean and absolute mean differences in border positions between the automated and manual segmentation for RPD of −0.75 ± 1.99 pixels (−2.92 ± 7.74 μm) and 1.53 ± 1.47 pixels (5.97 ± 5.74 μm), respectively. Additionally, in comparison to segmentation based only on gradient (Table 2), the algorithm produces results with much reduced variance.

Although there have been many studies characterizing the phenotype of non-neovascular AMD^12,28–31^; and although we and other groups have also developed algorithms for the segmentation of non-neovascular AMD lesions^32–34^, so far, there has been only one report to our knowledge of an algorithm for separate segmentation of RPD, RPE, and regular drusen on SD-OCT images, our group’s previous work^14^. However, significant segmentation errors were observed. In this study, we show once again that RPD and regular drusen can be separately segmented on SD-OCT images to allow the distribution and progression of these lesions to be studied separately. To the best of our knowledge, this is the first report of a validated algorithm for the automated segmentation of the retinal layers as well as both RPD and regular drusen separately on SD-OCT. The Deep Learning-Shortest Path strategy used for the segmentation of RPD and drusen is also shown to be generalizable to the segmentation of all layers of the retina.

In conclusion, in this study, an automated shortest path approach was developed utilizing deep learning convolutional neural networks to segment retinal layers associated with non-neovascular AMD. RPD and regular drusen were separately segmented on SD-OCT images to allow the distribution and progression of these lesions to be studied separately, and the approach is generalizable to multiple retina layer segmentation. In the future, this algorithm will be used to help evaluate a prospective dataset of 1000 patients showcasing drusen and RPD. With this inclusion of new data with accurate manual segmentation training data, the limitations of the pilot dataset used to develop the DL-SP algorithm can be addressed, leading to more accurate and generalizable automated segmentation.

## Data Availability

The image data utilized in this study are not publicly available due to the patients’ privacy and the violation of informed consent.
